# International medical learners and their adjustment after returning to their countries of origin: a qualitative study

**DOI:** 10.1186/s12909-024-05702-w

**Published:** 2024-07-05

**Authors:** Itthipon Wongprom, Onlak Ruangsomboon, Jikai Huang, Abbas Ghavam-Rassoul

**Affiliations:** 1 Department of Family Medicine, Faculty of Medicine Ramathibodi Hospital, Mahidol University, Rama VI Street, Ratchtevi District, Bangkok, 10400 Thailand; 2 Department of Emergency Medicine, Faculty of Medicine Siriraj Hospital, Mahidol University, 2 Wanglang Road, Bangkok Noi, Bangkok, 10700 Thailand; 3 Department of Nursing, Toronto General Hospital, University Health Network, 190 Elizabeth St, Toronto, ON M5G 2C4 Canada; 4 St. Michael’s Hospital, Unity Health,Toronto, Canada and Department of Family and Community Medicine, Temerty Faculty of Medicine, University of Toronto, 500 University Ave, 5th Floor, Toronto, ON M5G 1V7 Canada

**Keywords:** Repatriation, Readjustment, International medical learners, Coping strategies, Reverse culture shock

## Abstract

**Introduction:**

International medical trainees, including residents and fellows, must cope with many challenges, such as differences in cultural hierarchical systems, languages, and acceptance. Nonetheless, the need for adjustment perpetuates even after training is completed abroad. When some international trainees return to their countries of origin, they continue to face adjustment challenges due to reverse culture shock. Others must make many further readjustments. This study presents an exploration of the adjustment and coping strategies of international medical learners after returning to their countries of origin upon completion of their programs.

**Method:**

This study employed a qualitative approach grounded in interpretivism and utilised inductive thematic analysis following Braun and Clarke’s method. Semi-structured, in-depth individual interviews were employed to explore the participants’ coping strategies. Participants included international medical learners who were (1) international medical graduates who had already returned to their countries of origin, (2) non-Canadian citizens or nonpermanent residents by the start of the programs, and (3) previously enrolled in a residency or fellowship training programme at the University of Toronto, Ontario, Canada.

**Results:**

Seventeen participants were included. Three main themes and seven subthemes were created from the analysis and are represented by the Ice Skater Landing Model. According to this model, there are three main forces in coping processes upon returning home: driving, stabilising, and situational forces. The sum and interaction of these forces impact the readjustment process.

**Conclusion:**

International medical learners who have trained abroad and returned to their countries of origin often struggle with readjustment. An equilibrium between the driving and stabilising forces is crucial for a smooth transition. The findings of this study can help stakeholders better understand coping processes. As healthy coping processes are related to job satisfaction and retention, efforts to support and shorten repatriation adjustment are worthwhile.

## Introduction

International medical learners are typically physicians who pursue further training, such as a residency or fellowship training, in a country other than their country of origin. These learners play an essential role in Canada’s health care system. In Canada, in 2021, 4,376 international medical graduates were enrolled in residency and fellowship training programs [1]. International residents and fellows come from different backgrounds. In 2010, 20% of the residents enrolled at the University of Toronto were International Medical Graduates, defined as those who received their medical degrees from schools outside the U.S. and Canada; the majority identified themselves as having white ethnicity, while other ethnicities included South Indian (17%), Chinese (14%), Arab (6%), and others (19%) [2].

In the context of cultural adjustment to destination countries, international medical trainees must cope with challenges that are linked to their profession and practice, including differences in cultural hierarchical systems, language, fear of rejection, and concern about acceptance [3–5]. Additionally, adaptation to the practice of patient-centred care, which requires a deep understanding of cultural nuances and patient expectations, is particularly important [6]. These challenges stem from the switch of cultural paradigms in which learners are immersed when arriving in Canada. Considering the nature of stressors, coping strategies vary. For example, the stresses related to professional work could be mitigated by implementing effective orientation processes and institutional support, while personal stresses, such as feelings of isolation, can be managed by early detection and promoting social interaction. Additionally, it is argued that international students cope with cultural differences by balancing their educational goals and well-being [7]. Implementing pretraining preparation sessions is considered a helpful strategy in language and cultural domains [8, 9]. Newly acquired competencies could fuel international learners’ confidence and enhance their coping strategies. For instance, as parts of Canadian Medical Education Directives for Specialists (CanMEDs) framework, communication skills and professionalism can lay a solid foundation for these new specialists working in a new environment [10, 11]. Resilience building is also widely mentioned in the literature, with a focus on early-career and experienced physicians [12, 13]. Furthermore, some adjustment strategies learned and used during their training abroad, such as connecting with students’ cultural communities, taking language courses, and cultivating an optimistic mentality, may increase their reflective practice skills and resilience [7, 14–16].

Despite the preparation received during their training abroad, the need for adjustment persists even after returning to their countries of origin. A recent study revealed that international students face several poststudy challenges, including an uncertain future, immigration policies and reverse culture shock [17]. The phenomenon of reverse culture shock, characterised by difficulties in readjusting to one’s native culture, poses significant challenges for returning medical trainees [18–20]. In the medical field, this adjustment period is further compounded by differences in practice settings, work cultures, and the availability of medical resources, which can impede the seamless application of newly acquired knowledge and skills [21, 22]. In addressing these challenges, the literature suggests various coping strategies tailored to the unique needs of medical professionals. For instance, Adler proposed four distinct coping strategies—resocialise, proactive, rebellious, and passive—to facilitate readjustment periods [23]. Meanwhile, others argue that a positive sense of accomplishment, such as a professional identity, also helps during the coping period [11]. In business sectors, the literature suggests that time, mentorship, work autonomy, self-efficacy, and social status changes affect readjustment after returning from international training [24].

In medical education, limited attention has been given to the experiences of international medical learners as they transition back to their countries of origin following the completion of their programs. This qualitative study is intended to fill this gap through the examination of the coping strategies employed by international medical learners to navigate the challenges of readjusting to their countries of origin postrepatriation. Additionally, the aim of the study was to identify and clarify the challenges encountered by these learners during their training that may influence their adjustment upon returning home.

## Methods

### Study design

This study was conducted using an interpretative approach to qualitative research grounded in interpretivism and utilised inductive thematic analysis, following Braun and Clarke’s method, to explore how international medical learners cope with repatriation [25, 26]. In the exploration of how international medical learners cope with repatriation, a qualitative research method is essential to fully understand participants’ complex and diverse experiences. Interpretivism offers an appropriate framework because it focuses on the idea that reality is socially constructed and subjective. Interpretivism emphasises understanding the context and meanings individuals or groups attribute to their experiences. This approach allows in-depth exploration of participants’ repatriation experiences [27–29]. Additionally, a constructivist ontology and epistemology were adopted, acknowledging individuals’ subjective construction of reality and knowledge and emphasising the importance of understanding participants’ interpretations and meanings. This study received ethical approval from the Health Sciences Research Ethics Board, University of Toronto, Canada, on November 9th, 2022.

### Settings/participants

The participants included international medical learners who met the following criteria: (1) international medical graduates from any countries who have already returned to their countries of origin, (2) non-Canadian citizens or nonpermanent residents at the start of the programme, (3) previously enrolled in a residency or fellowship training programme at the University of Toronto during the 2017–2022 period. The participants’ countries of origin were not considered during the recruitment process. Participants were recruited using purposeful sampling and the snowball technique. After obtaining permission and contact information from the University of Toronto Postgraduate Medical Education Office, a brief invitation was sent via email to the learners and key contact persons, such as programme directors, chief residents or fellows, and some learners known to the investigators. Participants could refer other potential participants to the study, to whom detailed invitations were sent via email. The recruitment and interviews ended when the data were saturated. No incentives were given to the participants.

### Data collection and processing

Semistructured interviews based on a predefined interview guide were used as the primary means of data collection. The interview guide was developed specifically for this study based on relevant items guided by a comprehensive review of the literature. It was then refined to ensure alignment with the study objectives. Written informed consent was obtained before the interviews, and participants were encouraged to answer questions according to their comfort level. The interviews could be terminated at any time by the participants. The primary investigator, IW, conducted the interviews from October 2022 to April 2023. All interviews were performed online and recorded using the Zoom application. Participants selected the interview locations and times according to their preferences to ensure their comfort level during the interviews and for participants’ convenience.

The information collected in the interviews included the participants’ gender, age, primary language, ethnicity, speciality, country where their medical degree was obtained, programme of study in Canada, years in Canada, and experiences during and after training in Canada. Transcriptions were reviewed, and identifiable data, including countries of origin, names, and programs of studies, were extracted from the transcribed interviews.

### Data analysis

Thematic analysis was selected as the methodology for data analysis because it allows for identifying and interpreting patterns within the data, thereby providing insights into the participants’ experiences and perspectives [25]. Two independent researchers (IW and OR) performed the coding process by generating initial codes from the verbatim transcripts, followed by organising them into preliminary themes based on identified patterns. These themes underwent refinement through team discussion and were validated by revisiting the original data for alignment with participants’ narratives. If a consensus could not be reached, conflicts were resolved by investigator triangulation. Ultimately, a final set of themes was synthesised. We used NVivo to manage and share transcribed notes (QSR NVivo version 12, 2018). We ensured that the participants and their programs were deidentified in all parts of the analysis and the completed study reports.

## Results

**Fig. 1:**
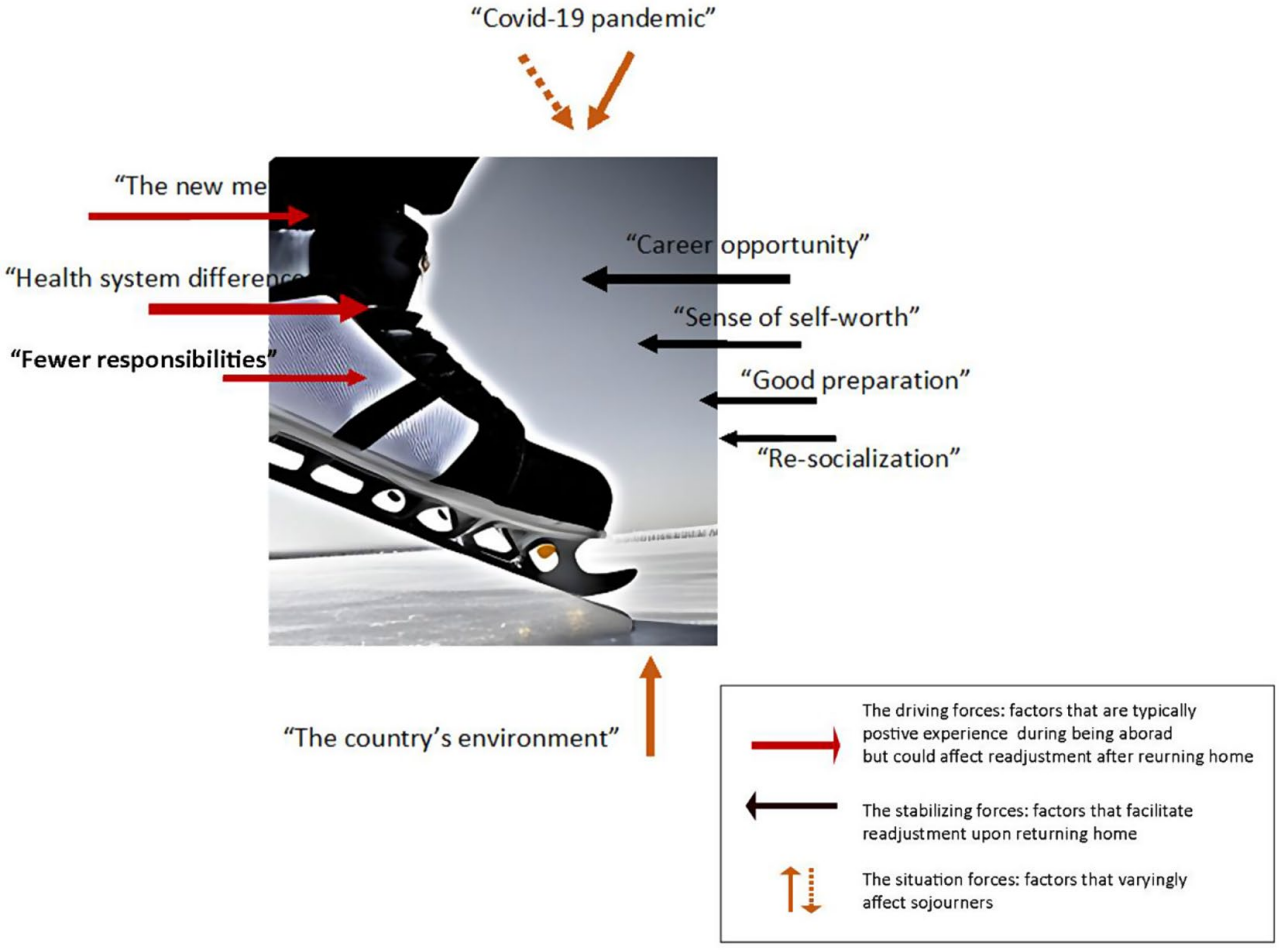
“Ice Skater Landing Model*”*

The interviews were conducted from October 2022 to April 2023, when data saturation was observed. A thematic analysis revealed three main created themes and seven subthemes, as illustrated in Fig. 1. Participants also voiced some suggestions that could be beneficial for future trainees.

### Participants

Seventeen participants were included, and their interviews were analysed. The participants were from 7 countries in four regions: South America, the Middle East, South Asia, and Southeast Asia. Among the seven countries, four are high-income, two are lower-middle-income, and one is an upper-middle-income country. 65% of participants were from middle-income countries. Among the participants, the time spent studying abroad varied from 1 to 3 years, with an average of 1.7 years. All participants had finished residency training in their countries of origin before attending a fellowship training programme, either a clinical or academic fellowship, in Toronto, Canada.

### Created themes: an ice skater landing model

The data analysis revealed three created themes and seven subthemes. The three main themes are (1) the factors that pose challenges to readjustment, (2) the factors that help readjustment, and (3) the factors that vary widely. To visually encapsulate the created themes, we propose the Ice Skater Landing Model, depicted in Fig. 1. The Ice Skater Landing Model illustrates a skater landing and the relevant stabilising and driving forces. Each created theme was represented by a force interacting with the skater. Metaphorically, a stable and good landing results from balancing the driving forces, such as speed and acceleration, and the stabilising forces, such as ice rink friction. On the contrary, if the skater is unbalanced, skaters may fall on their backs or knees depending on the direction of overcoming the force.

Similarly, there are three main forces in the coping processes of international medical learners who adjust themselves upon returning home: driving, stabilising, and situational forces. The sum and interaction of these forces impact their readjustment processes. For example, learners might face a challenge after returning home if a driving force is significant and a stabilising force is less powerful. Conversely, readjustment processes will be less difficult if a stabilising force counters another force in a balanced fashion. Depending on individual circumstances, situational forces represent additional factors that can influence the readjustment process. In certain instances, these forces can play a negative, neutral, or positive role in the readjustment process.

### The first theme: the factors that posed challenges to readjustment, the driving forces

Participants’ quotes depict elements commonly associated with positive experiences during overseas training, but these factors may pose challenges to readjustment after repatriation. These factors can be viewed as driving forces that push skaters to move forward. An analogy can be drawn to skaters propelled forward by force, which enhances their speed and progress. However, if balance is not maintained or the landing is poor, this force can lead to falls. Metaphorically, the driving force is supposed to make participants advance in their careers, as they are equipped with more skills and competencies and are immersed in positive experiences while studying abroad. However, repatriation following an extended period abroad presents formidable challenges stemming from several factors. Upon returning to their countries of origin, participants often encounter disparities in resources and systems. The manifestation can be in various aspects, such as technology, health care accessibility, and public services, necessitating a recalibration of potentially less sophisticated infrastructure. Additionally, the cultural landscape may no longer resonate with the enriched perspectives acquired abroad, further complicating the readjustment process.

The driving forces comprise three created subthemes, or forces: “the new me”, “health service system”, and “fewer responsibilities”.

• “The new me”.

During training in Canada, some international medical learners reported acquiring new attitudes or perspectives, such as having more self-awareness and the concept of equality/equity. These attitudes relate to either training programmes or spending time living abroad.

*“When you see something outside [the country of origin], like in the States or Canada, everyone is…like… equal, no that much of seniority or something like that. Focusing on just the system or the patient care is good for everyone.” [P011]*.

Although some learners appreciate these changes and see them as a part of transformative changes, others feel these shifts hinder resettlement upon returning home. They face difficulties fitting into local contexts, lifestyles or cultures on several levels. They know that the contexts are the same as before leaving home; nonetheless, it is difficult for them to live or work the way they used to, given that they have transformed into their new selves.

*“And when you come back, you are not the same people and something you really ….like….you lost your proper pace. I feel completely different because I missed a lot of [name of the country] when I left here. And now here, sometimes with my same colleagues, I say…. maybe I don’t belong to this place anymore. It’s difficult for me.” [P009]*.

“Health service system”.

The differences in the health service systems between Canada and the participants’ home countries play a significant role in their readjustment. The participants identified several strengths of the Canadian health care system, including advanced resources, comprehensive support teams, and efficient care processes. While these advantages benefit learners’ training and enhance their medical proficiency, they pose challenges when participants return to their countries of origin, where limited resources and less support necessitate adaptation and improvisation.

*“You’re talking about developing country and a developed country. So, there is certainly a huge difference, especially from the perspective of access to health care in [name of the country]. The public health care system is not very well developed, and Canada, it’s a huge very. very impressive public health care system.”* [P002].

- “Fewer responsibilities”.

Some participants have fewer responsibilities during their training abroad than before departing; they have finished residency training in their home countries and have been practising their specialities for a while. Before going to Canada, they had some work experience and responsibilities, such as teaching and administration. However, in Canada, they dedicated themselves entirely to their training, with minimal additional commitments. As full-time fellows, they were spared from the administrative burdens typically associated with their roles in their home countries. Moreover, residing in a foreign land allowed them to explore unfamiliar territories and immerse themselves in new experiences. Hence, this period often feels like a temporary reprieve for them, a chance to recharge before diving back into their professional duties as specialists.

However, upon returning to their countries of origin, these individuals often face many responsibilities on various fronts; they must perform administrative duties within hospital settings, attend to financial matters, and prioritise their career development while balancing the demands of their specialised roles. This transition from a focused training period to the complexities of professional life at home underscores the significant adjustments required as they resume their roles with heightened responsibilities and expectations.

“Previously, I took on a lot of responsibilities, like university and extra work and things like that. And in Toronto, an advantage was that I did not work weekends most of the time. Sure, there were the calls, but that was… like once a month, every two months. So, most of the time, I had time. So, I could listen to podcasts and read books.”[P017].

*“I only have one year, and I want to experience as many places as possible. So, I try to go out on every single vacation I have.”* [P001].

### The second theme: the factors that support international medical learners’ readjustment, the stabilising forces

The second central theme is the factors that support international medical learners’ readjustment. Participants perceive that these factors contribute to facilitating their readjustment upon repatriation. Stabilising forces were proposed to represent this theme. These are the counterforces that help the skaters land and stop smoothly without falling. These forces can occur either before or after international learners return to their country of origin. There are four created subthemes representing these forces: “good preparation”, “career opportunity”, “sense of self-worth”, and “resocialisation”.

• “Career opportunity”.

The broadest stabilising force is expanding career opportunities after returning to their countries of origin. Most participants felt that they gained considerable knowledge and skills from their training abroad, resulting in more confidence in practice, education, and administration. As they finish further training in their fields, their careers advance. Some are involved in establishing innovative services or educational programmes as pioneers, and others have more job opportunities than ever before. This phenomenon positively impacts their readjustment, mitigating the challenges posed by differences in the health service system.

*“I have been here for just a month, and I feel people are…have more respect for you, and they listen to you more. They will hear your suggestions into your knowledge, your contributions to medical education.”* [P006].

• “Good preparation”.

This subtheme was created from the analysis and the participants’ direct answers when they were asked about their suggestions for future returnees. They value proper preparation prior to returning home, not only regarding work-related arrangements, such as getting to know their new workplace, but also regarding mental preparation, such as anticipating common challenges and changes.

*“Hmm! I would say before going back, just knowing what position you gonna be taking is important. Because you’re gonna build up toward that’s position, to fill that position as, in terms of requirement or job description, things that you want to develop personally or professionally to fill in that position.” [P005]*.

Additionally, participants stress the importance of being aware that adjustment takes time; this mentality prepares them to put fewer unnecessary burdens on their shoulders during the adjustment process.

*“I’ve been expecting to encounter these challenges, so it is more of managing expectations, I guess, similar to doing palliative care for our patients. As long as I know that I will be encountering these challenges at some point, you just try to do what you do, and then you will accept the limitations that are in place.” [P012]*.

• “Sense of self-worth"

This subtheme represents a force that counters the driving forces by empowering returnees to embrace their successes and endurance during training. They feel that they have survived an arduous journey and earned monumental achievements after they finished high-level training at a renowned educational institute in a developed country.

*“The sense of creating better good for larger population was something that I gained from this fellowship.” [P002]*.

• “Resocialisation”.

Nearly all participants reported the positive effect of support from their families and social networks upon repatriation. While away in Canada, international learners typically depart from their usual social circles, such as families, friends, and colleagues. Returning home provides opportunities to reconnect to their familiar environment and supportive social networks.

*“Our friends and our other social circles, going back to work was also coming back to a familiar environment, specifically just because it was the same hospital I was trained in before going to the fellowship. So I had a very warm welcoming from my colleagues at the workplace.” [P007]*.

### The third theme: the factors that affect varyingly, the situational forces

Finally, the third main theme reflects factors that affect participants’ readjustment differently, depending on the international medical learner context. This theme could be viewed as situational forces. These forces are, metaphorically, the factors that indirectly impact the skater’s landing but have some effect on international learners’ changes and experiences. Some of these forces affect each individual to varying degrees, depending on their context. These factors are categorised into two subthemes: “experience during the COVID-19 pandemic” and “the country’s environment”.

• " experience during the COVID-19 pandemic “.

The first subtheme is a unique scenario in this study, as all participants stayed in Canada for their training during the COVID-19 pandemic. Some participants expressed a sense of relief after the pandemic subsided, while some felt safer in Canada than in their countries of origin, where the resources were more limited. The pandemic affected their professional and personal lives in some ways; some cited it as a profound stress. There are mixed responses from the analysis, none of which directly impact the readjustment processes. Nonetheless, this force could amplify some stabilising forces, such as resocialisation and a sense of self-worth, as participants feel more rewarded after reuniting with their families and surviving increased hardship during the pandemic.

*“Because, as I said, I was alone. I don’t have too worried about my families. I don’t want have to worry that I’ve got to spread it to my…. to transmitted to my family members. “[P001]*.

*“Of course, socially we couldn’t interact that much more. That was a bit of a shame where we couldn’t really interact and have that much kind of social interaction with our fellows. Of course, we tried by much smaller groups and of course, having that social life in Toronto, that took a hit as well because a lot of things were shut down.” [P015]*.

• " the country’s environment”.

The second subtheme includes any aspects of the environment other than the health service system, such as weather, transportation, infrastructure, social norms, and culture. This force can either contribute to the driving forces, making readjustment processes more challenging or amplifying the stabilising forces, resulting in a more effortless adjustment. In some cases, this force remains neutral. For example, participants from a country with a social system similar to that of Canada did not feel that they needed to adapt much. However, some mentioned that traffic-related issues in their countries of origin initially made their resettlement more difficult. The magnitude of the problems also varies and depends on the individual’s context. Minor inconveniences, such as changes in weather or routine lifestyles, could eventually be managed.

*“And then traffic, Canada really has less traffic compared to here. And the public transport is better in Canada also. So it was really convenient to live there as a fellow because time was really reduced for travel here. In the [name of the country], it’s like one and a half hours to go to the hospital on some days and one and a half to go back.” [P013]*.

*“All challenges that you need to adjust yourself. I didn’t stay in Canada for that long because [name of the country] …. because I think Toronto is a very cosmopolitan city. It’s quite similar to [name of the country], actually. I mean, we are both English speaking kind of cities, large Asian population. The culture isn’t that much different, so wasn’t really much different.” [P015]*.

## Discussion

The analysis adopts the Ice Skater Landing Model, which is used to explain how international medical learners cope with readjustment after returning to their countries of origin. A balance of driving and stabilising forces, along with situational forces, contributes to a smooth transition and a good adjustment. The results and created subthemes shed light on detailed readjustment processes. Furthermore, they could be addressed and understood to prevent reverse culture shock.

The results of prior studies are consistent with the analysis proposed in this study. In Adler’s re-entry coping model, external validation and change awareness are essential in returnees’ adjustment [23]. The two mentioned forces, good preparation and career opportunities, are consistent with external validation and change awareness. Previous literature also argues that social support through social reconnection is vital for dealing with reverse culture shock by returnees [19, 30]. Furthermore, mental preparation, a part of the “good preparation force”, is a crucial factor influencing the readjustment process after returning home. For example, paediatric residents returning from a rotation abroad used expectation management to reduce pressure on themselves [31]. Similarly, accuracy in work expectations affects repatriation adjustment among returning workers to Spanish international corporations [24].

### Implication

Although some factors, such as the environment or family support, are unmodifiable, others can be targeted for intervention. The literature shows that comprehensive bridging programmes could help international medical learners adjust themselves before and during training [30, 32, 33]. The created subthemes presented in our analysis suggest that backward bridging programmes that provide better preparation for incoming returnees could be helpful. This operation could be performed from the training programmes’ side if the training institutes and leaders value their former trainees’ success as an excellent training outcome. In contrast, the funding institutes could also do the same if they would like their returning faculty members to experience a smooth transition, leading to greater job satisfaction and retention [24, 33]. Many issues and challenges could be overcome by the mentioned programmes through adequate preparation. This type of programme can cover a wide range of actions, from emphasising an ongoing network from both sides throughout the training, anticipatory guidance about what should be expected, and enhancing reflective learning to shape learners’ attitudes toward their self-accomplishments or self-worth and their transformation. Furthermore, these subthemes could be helpful inputs for curriculum development, as many trainees require reflective skills and mentoring during training programmes.

### Limitations

It is worth mentioning that this study’s results should be interpreted with caution for the following reasons: time and country. First, as proposed by previous studies, sufficient time after returning home is considered an essential factor in readjustment [19, 24, 34]. This aspect was not addressed in the results of this study, which is most likely because our analysis was framed upon how the readjustment is processed, regardless of the re-entry phases. Additionally, most participants were interviewed shortly after they resumed working, approximately 3–6 months after repatriation. Second, most participants were from middle-income countries before joining the training programme in a high-income country like Canada. Contextual differences, such as situational forces, could affect readjustment processes and should also be considered when generalising our results to other populations. Finally, there were no participants from Europe or Africa. Including perspectives and experiences from these regions might yield different results.

Nonetheless, this study has several strengths that should be mentioned. First, the study included health care learners from various regions of the world; therefore, it portrayed a broad picture of readjustment among diverse international medical learners. Furthermore, the COVID-19 pandemic is a unique factor that may have confounded readjustment processes. Although not this study’s primary objective, the effect of the pandemic was demonstrated in the results and could be explored further.

### Future research

In future studies, further attention could be given to how the readjustment process can be shortened and made more efficacious. It would be beneficial for both institutes and returnees if standardised, effective tools were developed to accelerate the processes. It is also imperative to explore perspectives from funding organisations or institutes, as they are the key stakeholders.

## Conclusion

International medical learners trained abroad often struggle with readjustment upon returning to their countries of origin. This qualitative study proposes the “Ice Skater Landing Model” to explain how individuals cope during readjustment. An equilibrium of driving and stabilising forces is crucial to achieve a smooth transition. Good preparation and re-socialisation are the essential elements of stabilising forces, helping returnees better cope. The findings of this study can help stakeholders, such as faculty leaders and training programme directors, better understand coping processes. As healthy coping processes are related to job satisfaction and retention, efforts to support and shorten repatriation adjustment are worthwhile. We propose that a comprehensive bridging programme would benefit all stakeholders. Further research is warranted to explore the models and effectiveness of these preparation programmes.

## Data Availability

The datasets generated and/or analysed during the current study are not publicly available due to participants’ privacy and the sensitive nature of collected data but are available from the corresponding author on reasonable request.
